# Enhancing West Nile Virus Surveillance, United States

**DOI:** 10.3201/eid1006.030457

**Published:** 2004-06

**Authors:** John S. Brownstein, Theodore R. Holford, Durland Fish

**Affiliations:** * Yale University School of Medicine, New Haven, Connecticut, USA

**Keywords:** Bayesian Method, Disease Vectors, Encephalitis, Viral, Geographic Information Systems, Markov Chain Monte Carlo Method, Sentinel Surveillance, Topography, Medical, West Nile virus

## Abstract

We provide a method for constructing a county-level West Nile virus risk map to serve as an early warning system for human cases. We also demonstrate that mosquito surveillance is a more accurate predictor of human risk than monitoring dead and infected wild birds.

The introduction of West Nile virus (WNV) to the Western Hemisphere resulted in a human epidemic in New York City during 1999 (1). By 2002, WNV had spread to 44 states and the District of Columbia, with a total of 4,156 human cases of infection reported by the Centers for Disease Control and Prevention (CDC). Although a nationwide human surveillance system has been established, passive surveillance data are problematic because of variability in disease reporting. The inaccuracies in disease reporting are compounded by random variability inherent in estimating disease incidence rates, a fact that makes interpreting a risk map based on raw data difficult (2). Accounting for these issues should allow for a more precise delineation of spatial risk patterns and for improved targeting of limited prevention resources earlier in the transmission season. In addition to human cases, risk for WNV can be assessed by nonhuman surveillance systems, including infected birds and mosquitoes (3). However, these systems have not been statistically compared for their predictive ability of human risk. A quantitative assessment of the value of the nonhuman surveillance systems would also help direct resources for WNV surveillance. We provide a statistical method to estimate an accurate early assessment of human risk and to determine the predictive capabilities of nonhuman surveillance systems.

## The Study

### Human Surveillance Model

Human case data were taken from the weekly U.S. Geological Survey West Nile maps for the 2003 transmission season based on county-level data provided by ArboNet through voluntary reporting by state and local health officials to CDC (4). The case numbers comprise reports of mild West Nile fever as well as the more severe West Nile meningitis or encephalitis. Crude county-specific incidence rates were calculated by using the Census 2000 county population totals.

We created a human risk map for WNV based on the crude human incidence early in the transmission season, on August 13, 2003. A disease map that displays observed human incidence will show not only spatial variation in risk but also random variation resulting from low case numbers relative to the base populations. Removing random noise permits improved estimates of disease risk (2). We have approached this procedure by finding the estimates of expected incidence from a conditional autoregressive model (5,6). The model helps remove random variation based on the premise that contiguous regions tend to have similar disease risks, when compared to regions that are far apart. We applied the conditional autoregressive model to calculate expected WNV incidence rates (Appendix).

The first step was to identify the adjacent neighbors of each county by using a geographic information system (GIS, ArcView 3.2, ESRI, Redlands, CA). A data file that included the number of cases, total population, and number and names of neighboring counties for each county was then generated in SAS (SAS Institute Inc., Cary, NC). The file was imported into WinBUGS v1.4 (Imperial College, St. Mary's, UK; and Medical Research Council, Cambridge, UK). This software implements a simulation process to estimate model parameters, including improved estimates of WNV incidence rates. These estimates were then brought back into GIS to display the human WNV risk map.

To verify the method's potential use as an early warning system for human risk, we calculated the validity of the model-estimated risk map versus the raw incidence map from August 13 for predicting the case distribution for October 1, 2003. The two time points represent an ≈14-fold increase in total cases, from 399 to 5,685. For each of the three disease maps, counties were grouped into high- and low-risk classes on the basis of WNV incidence. High risk was defined as human incidence >1 case per 1 million population for the August 13 maps and 1 case per 100,000 for the October 1 map, findings that reflect the change in risk over time. The sensitivity of the method for predicting risk was calculated as the proportion of high-risk counties on October 1 that was correctly identified as such by the model-estimated August 13 risk map. Similarly, specificity was defined as the proportion of low-risk counties on October 1 that was correctly identified as such by the modeled risk map. The sensitivity and specificity values were compared to those obtained when the raw August 13 incidence map was used to predict risk on October 1. Measure of agreement between risk classes of the August 13 map and the October 1 map was assessed by the k statistic, which accounts for the degree of overlap expected by chance alone; k has a range of 0 to 1; values of <0.4 represent poor agreement (7).

### Nonhuman Surveillance Model

We assessed the quantitative predictive ability of the nonhuman surveillance systems by fitting a regression model to the rate of WNV human cases for counties with the final USGS maps for the 2002 season (4). The model includes covariates for the number of virus-positive tissue samples from dead and diseased wild birds and virus-positive mosquito pools, both provided by state health officials at the county level (Appendix). Each covariate was considered together and separately to determine its contribution for predicting WNV incidence. The model was fitted by using GENMOD in SAS (SAS Institute). The contribution of nonhuman surveillance systems to variability in human risk was determined by calculating the proportion of the deviance explained (R^2^).

## Conclusions

**Figure 1:**
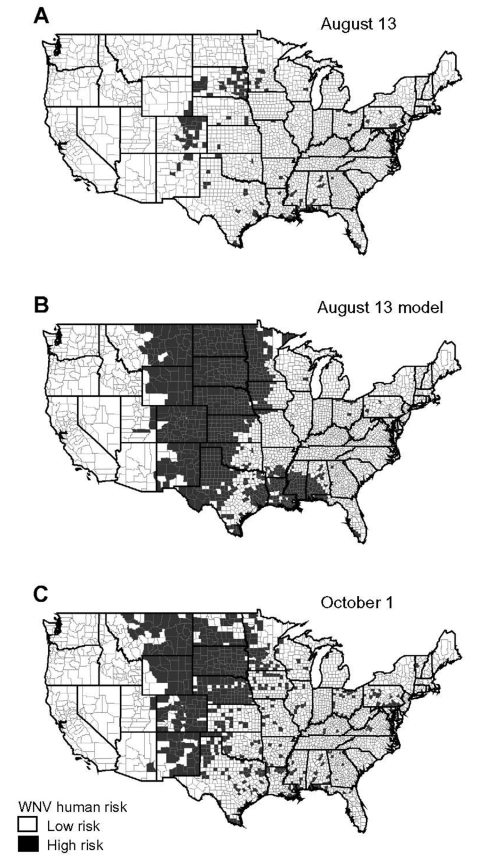
A) Human incidence map for West Nile virus (WNV) early in the transmission season, 2003, based on raw data. Incidence rates were calculated by using the number of new human cases of WNV per county through August 13, 2003, reported to the ArboNet surveillance network. High risk is defined as incidence >1 case per 1 million inhabitants. B) Model-estimated human incidence map for WNV in 2003. Expected risk was derived from the observed incidence rates from August 13, 2003. High risk is defined as incidence >1 case per million persons. C) Observed human risk for WNV late in the transmission season, 2003. Incidence rates were calculated by using the number of new human cases of WNV per county through October 1, 2003. High risk is defined as incidence >1 case per 100,000. This risk surface served to compare the predictive ability of the (A) raw versus (B) modeled early season disease maps.

The maps of Figure 1 show the raw county-specific incidence rates for August 13, 2003 (Figure 1A), the model-estimated risk for August 13 (Figure 1B), and the raw incidence rate on October 1 (Figure 1C). The model-estimated risk surface of August 13 displays a much larger area of high risk than the reported incidence map on the same date, with 930 high-risk counties compared to 128 counties (Figure 1A and 1B). The disease map for October 1 shows a similarly larger high-risk area, with 569 counties classified as high risk (Figure 1C).

The early warning capability of our model was evaluated by comparing the validity of the raw and modeled early season disease maps for predicting the case distribution late in the transmission season (October 1). The raw data on August 13 produced a sensitivity of 19.7% (112/569) for predicting high-risk counties on October 1. In contrast, application of the model allowed for 76.1% (433/569) of the October 1 high-risk counties to be predicted, yielding a fourfold increase. This increase in sensitivity did not have a comparable negative effect on specificity, which decreased from 100% to 80.4% (2,043/2,540). In addition, the August 13 model yielded good agreement with the October 1 data, as shown by a k statistic of 0.45 (95% confidence interval [CI] 0.42 to 0.49), whereas agreement was poor when the raw August 13 map with a k statistic of 0.27 (CI 0.23 to 0.31) was used. Accounting for confounding caused by age distribution of WNV patients could further improve overall validity of our model.

This method has the potential to be applied in real-time to identify high-risk counties before the major influx of cases during the transmission season. The model could enable control methods to be implemented early in the season as prevention efforts before the first human case. This time advantage could provide more effective disease prevention efforts.

**Figure 2:**
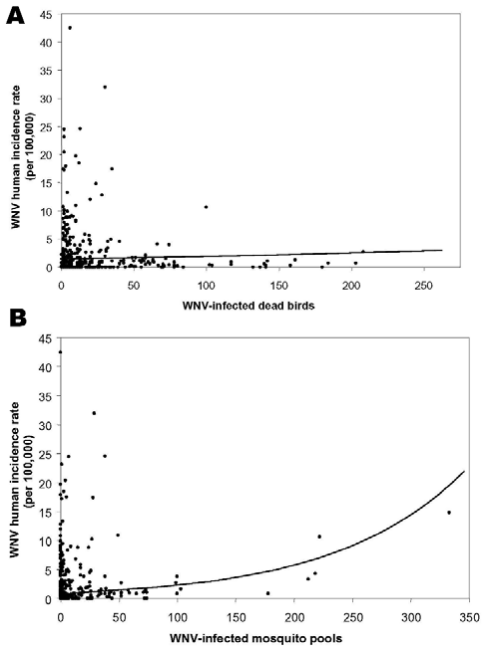
Plots of West Nile virus (WNV) incidence by collections of virus-positive dead birds (A) and virus-positive mosquito pools (B). Log linear models fit to both surveillance systems considered alone are displayed. WNV-infected dead birds explain 2.5% of the variation in human incidence (A), whereas WNV-infected mosquito pools explain 38% (B).

Risk modeling can also be used to effectively quantify the utility of nonhuman surveillance. Despite support for the use of bird surveillance as an early warning for WNV human risk (8–10), this system has not been statistically compared to active mosquito surveillance. The predictive ability of these surveillance systems for human risk was assessed by their inclusion as quantitative variables in a regression model. Although each variable alone was a significant predictor of human risk (χ^2^_bird_ = 138.0, p_bird_ < 0.0001; χ^2^_mosquito_ = 2,605.9, p_mosquito_ < 0.0001), the numbers of WNV-infected dead birds could only explain 2.5% of the deviance, whereas the number of WNV-positive mosquito pools explained 38%. Thus, quantitative mosquito data predict 15 times more of the variation in human cases than quantitative bird data do. The model with both covariates also explained 38% of the deviance by showing that bird data added proportionally less information about human risk (χ^2^_bird_ = 5.3, p_bird_=0.022; χ^2^_mosquito_ = 2,489.0, p_mosquito_ < 0.0001). Plots of the observed and fitted incidence rates, when compared to the covariate alone, showed a much stronger positive relationship between human WNV incidence and the number of WNV-positive mosquito pools than for WNV-positive dead birds (Figure 2).

Our finding that mosquito surveillance is more sensitive to human risk than bird surveillance can be explained by the fact that human infection in the natural WNV cycle is accidental (11–14). Because birds are the zoonotic reservoir host, a WNV-infected bird only indicates enzootic transmission. For human transmission to take place, mosquito species that can act as bridge vectors must be present in sufficient numbers. Therefore, because mosquitoes represent the link to human transmission, mosquito infection prevalence should more accurately predict human risk. Furthermore, once the important human vector species can be clearly identified, the predictive ability of mosquito surveillance should increase. Standard methods for collecting mosquito data applied uniformly would also greatly aid the interpretive value of these data. Our analysis has shown that active mosquito surveillance should be emphasized in WNV surveillance systems, as it is the most sensitive marker of human risk. Surveillance systems based entirely on dead bird reports lack sensitivity for early warning as well as crucial abundance data for targeting effective prevention efforts. Entomologic surveillance should continue to be the keystone for public health programs directed toward preventing WNV infections in humans.

In summary, disease surveillance and prevention efforts could benefit from enhanced risk mapping that draws from corrected human case data and a clear understanding of the predictive ability of nonhuman surveillance.

## Appendix

### Human Surveillance Model

Because the human West Nile virus (WNV) case number is low relative to the base population, it was assumed to have a Poisson distribution. Under general conditions, the Poisson provides a good description for the distribution of the numerator for an incidence rate (15). However, our model also allowed for the estimation of "extra-Poisson" variation in case it is also needed to provide an accurate description of these data.

The log linear model used for spatial smoothing assumed that the number of disease cases in the *i*-th county, *n_i_*, has a mean, *P_i_2_i_*, where *P_i_*, is the denominator for the rates and *2_i_* = exp [*a_0_* + *b_i_* + *h_i_*} where *a_0_* is the intercept, *b_i_* is the spatially correlated random variation with mean 0 and variance _
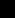
_, and *h_i_* the unstructured extra-Poisson variability with mean 0 and variance _
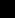
_. In addition, we assume that both the spatial and the unstructured variability have Gaussian distributions, which are independent in the latter case. On the other hand, the mean for the spatial component, conditional on the means for the contiguous neighbors, is


_
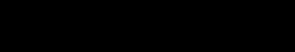
_


where *r_i_* is the number of neighbors for region *i*. The adjacent neighbors for each county were determined by using a geographic information system (GIS, ArcView 3.2, ESRI, Redlands, CA). Thus, the overall log linear model for the number of cases in the *i* county that incorporates both spatial correlation and unstructured variability is log *n_i_*_= log_ *P_i_*_+_ *b_i_*_+_ *h_i_*_+_ *a_0._*

The population size for county *i* (*P*_i_) was determined from the Census 2000 data.

Markov Chain Monte Carlo (MCMC) simulation methods were used to find Bayesian estimates of the model parameters as implemented in WinBUGS v1.4 (Imperial College and Medical Research Council) (16,17). Gamma prior distribution parameters were assumed for the variances of the Gaussian distributions, and a plot of the history of the simulation was used to determine the number of iterations required for the process to equilibrate. The approach provides improved estimates of county-specific rates that have been spatially smoothed.

In the MCMC method, parameters estimated from each step are used in turn to determine values for the next step; therefore, a good set of initial values is essential before gleaning the values that will be used in the estimation. To accomplish robust parameter estimates, an arbitrary set of values was chosen, and the number of successive steps taken to stabilize the simulations was noted, which is known as the burn-in. The burn-in period was determined through the use of two chains and the modified Gelman-Rubin convergence statistic. This statistic indicates the point at which the process stabilized by describing how well the chains overlap. Final estimates were obtained by using 1,000 iterations as the burn-in period, and the next 9,000 were used as the sample for deriving the Bayes estimates of the smoothed WNV incidence rates.

### Nonhuman Surveillance Model

**Figure A1:**
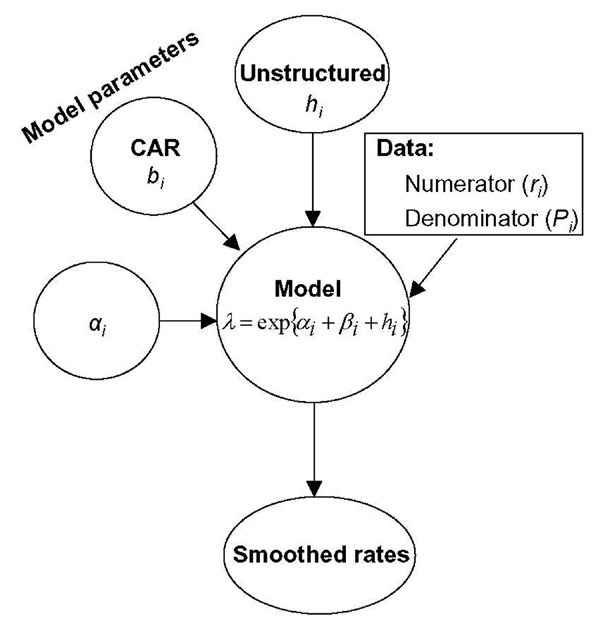
Diagram of the conditional autoregressive smoothing model.

The quantitative predictive ability of the nonhuman surveillance systems was assessed by once again fitting a log-linear model to the rate of WNV human cases. For this analysis, we instead used a maximum likelihood approach, in which we assumed a Poisson distribution for the number of cases, allowing for extra-Poisson variation by estimating the scale factor. In this model, log *n_i_* = log *P_i_* + *β_A_M_i_* + *β_M_A_i_* + *a_o_* where *P_i_* is the population offset, *A_i_* is avian mortality attributable to WNV, and *M_i_* is the number of virus-positive mosquito pools. The model was implemented by using GENMOD in SAS (SAS Institute Inc., Cary, NC). Only counties that submitted both mosquito and bird samples were included in the analysis (N = 382) (Figure A1).
